# Already current practice? A snapshot survey on doxycycline use for prevention of sexually transmitted infections in parts of the German MSM community

**DOI:** 10.1007/s15010-023-02086-9

**Published:** 2023-08-22

**Authors:** Daniel Hornuss, Philipp Mathé, Susanne Usadel, Stefan Zimmermann, Matthias Müller, Siegbert Rieg

**Affiliations:** 1 Division of Infectious Diseases, Department of Medicine II, Medical Center-University of Freiburg, Faculty of Medicine, University of Freiburg, Hugstetter Str. 55, 79106 Freiburg, Germany; 2 Department of Infection Medicine, Medical Service Centre Clotten, Freiburg, Germany; 3 Checkpoint Aidshilfe Freiburg e.V., Freiburg, Germany

**Keywords:** Doxycycline, Sexually transmitted infections, STI, Prophylaxis, PEP, PrEP

## Abstract

**Purpose:**

Recent studies point toward a potential benefit of doxycycline use for post-exposure prophylaxis (PEP) or pre-exposure prophylaxis (PrEP) to prevent sexually transmitted infections (STIs). Although prescribing doxycycline in a prophylactic intention is not generally recommended yet, we noticed an increasing number of inquiries from individuals within the LGBTQ community for doxycycline prescriptions.

**Methods:**

We conducted an anonymous online survey to evaluate the current extent of doxycycline use for PEP or PrEP within the LGBTQ community using REDCap electronic data capture tools. Participants gained access to the online survey through a QR code on posters in the premises of our STI outpatient department and at LGBTQ community-related events in the south-western region of Germany. Additional access was provided by a direct link shared on social media profiles for men having sex with men (MSM), transgender, and queers.

**Results:**

96 of 99 responses were eligible for analysis. Twenty-two participants (23%) indicated to have already used doxycycline for PEP and six participants (6%) used doxycycline for PrEP. The majority of participants used pills left over from previous doxycycline treatment. Forty percent of indicated modes of access were without a regular prescription, e.g., by provision from acquaintances (with or without healthcare profession) or by ordering online.

**Conclusion:**

Our study shows that the concept of doxycycline use for prevention of STIs is already well known and applied in the LGBTQ community. Further analysis, especially modeling studies, are needed to evaluate strategies aiming to reduce doxycycline intake (PEP/PrEP versus repeated targeted therapies) and improve sexual health outcomes within the community.

## Introduction

Sexually transmitted infections (STIs) remain a significant public health concern worldwide, affecting individuals across various sexual orientations and communities. In recent years, there has been a growing interest in exploring preventive strategies adapted to the needs of vulnerable groups, especially parts of the LGBTQ community (predominantly gay and bisexual men). Pre-exposure prophylaxis (PrEP) with doxycycline was shown to prevent the occurrence of syphilis in group of 30 men having sex with men (MSM) with high risk for STIs [1]. In an open-label study in MSM living in France, post-exposure prophylaxis (PEP) with doxycycline, taken within 24 h after sexual contact, reduced the risk of acquiring chlamydial infection or syphilis by 70% and 73%, respectively [2]. Data from the ongoing DOXYVAC trial, reported at the CROI 2023, seem to underline the efficiency of doxycycline-PEP with a combined reduction rate of 80% for infections with chlamydia or syphilis [3]. Moreover, another open-label trial showed a beneficial effect of doxycycline-PEP, taken within 72 h after sexual contact, on acquiring STIs (risk reduction 87% for syphilis, 88% for *Chlamydia trachomatis*, and 55% for *Neisseria gonorrhea*) in MSM on HIV-PrEP living in USA [4]. Based on these studies, the Australasian Society for HIV, Viral Hepatitis, and Sexual Health Medicine (ASHM) stated in an interim position statement that doxycycline-PEP could be useful for people with repeated bacterial STIs. The German Society on STIs (DSTIG) recommends the use of doxycycline for prophylaxis of STIs only in individualized cases [5, 6]. The limited evidence concerning potential risks of preventive use of doxycycline is contrasted by an increasing number of inquiries from individuals to prescribers regarding the use of doxycycline-PEP or PrEP—a trend we also noted in our STI outpatient clinic. Accordingly, recent studies have shown a broad interest in taking chemoprophylaxis for syphilis prevention [7].

## Methods

We conducted an anonymous online survey within the MSM community to elucidate to what extent doxycycline PrEP or PEP may already be used. Study data were collected and managed using REDCap electronic data capture tools [8]. Participants gained access to the online survey through a QR code on posters in the premises of our STI outpatient department and at LGBTQ community-related events in the region of Freiburg, a 230.000 inhabitant university city in Southwestern Germany. In addition, a direct link to the online survey was shared on social media profiles for MSM, transgender, and queers (romeo.com, GRINDR). The survey included questions concerning age range, area of residence by providing the first two figures of the postal code, and the use of doxycycline which was differentiated between never (I have never took doxycycline so far), for targeted therapy of STIs (e.g., chlamydia), and for PEP or for PrEP (which was defined as course of a single dose of doxycycline before a planned sexual contact or an ongoing course of more doses for prophylaxis). If doxycycline use as part of PEP or PrEP was indicated, further questions were asked about the frequency of courses with doxycycline-PEP or PrEP and how the medication was obtained. For analysis, we used descriptive statistics and Fisher’s exact test. The margin of error (95% confidence interval, 95%-CI) for survey responses was calculated with a standard deviation of 0.5 as described elsewhere [9]. Informed consent was not obtained in concordance with the Declaration of Helsinki and the regulations of our Institutional Review Board.

## Results

**Fig. 1:**
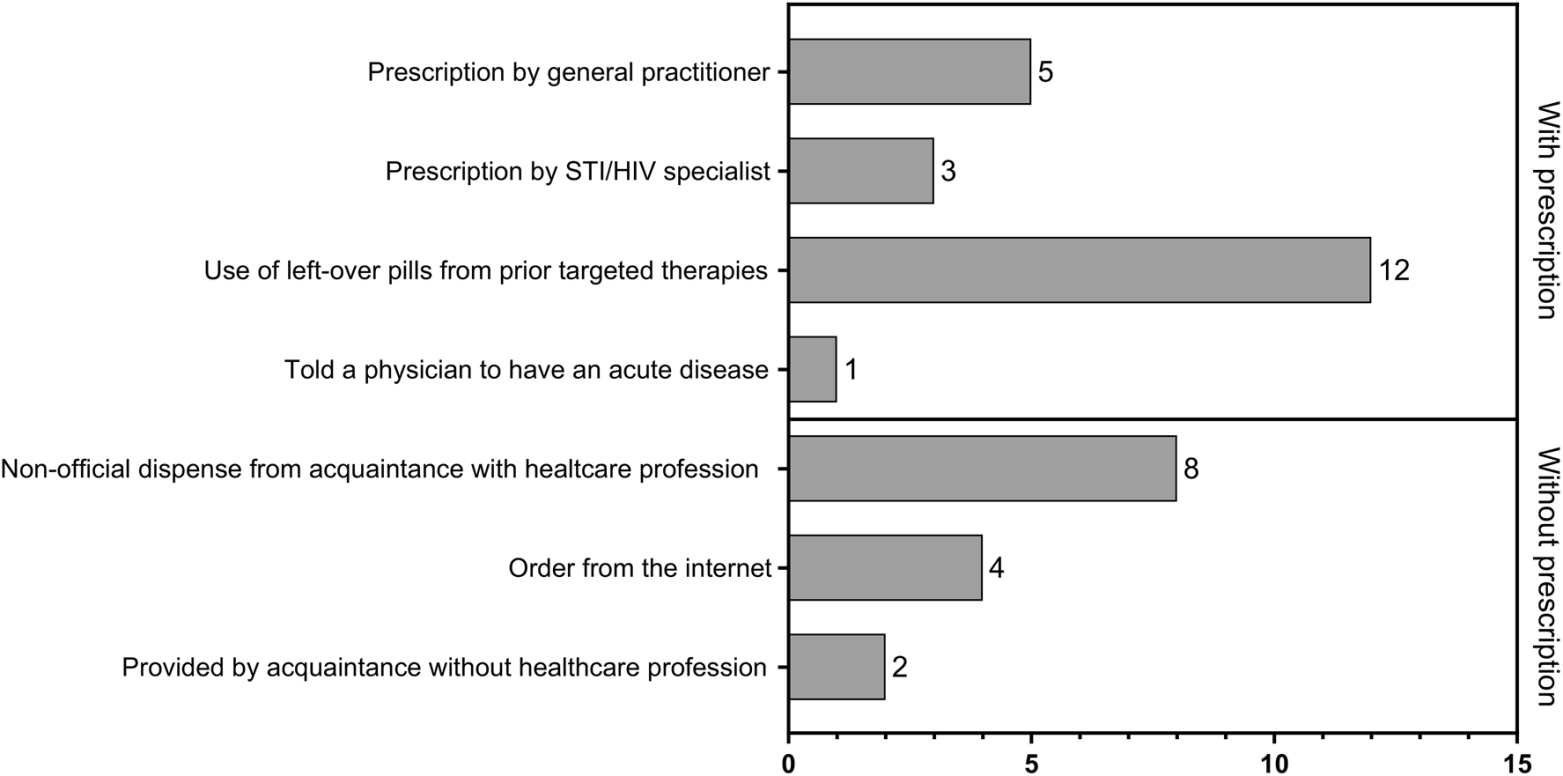
Modes of access to doxycycline for PEP or PrEP use

A total of 99 participants located in different catchment areas took part in the survey. Data of three participants were excluded due to non-conclusive responses. Seventy-six participants were located in the catchment area of Freiburg and 16 participants from Berlin. Seven participants were located in other catchment areas or did not provide information about the location. Participants were predominantly between 26 and 40 years old (median age range 31–35). Almost 55% of the participants had received doxycycline as part of a targeted therapy in the past (Table 1). Twenty-two participants indicated the use of doxycycline for PEP (23%, 95% CI 13–33%) and six participants already used doxycycline for PrEP (6%, 95% CI 0–16%). The total number of PEP or PrEP courses with doxycycline was predominantly less than 10. We found no significant differences in the use of doxycycline as PrEP (*p* = 0.819) or PEP (*p* = 0.747) between age groups, while the odds for a past doxycycline therapy significantly increases above 30 years of age (OR 5.3, *p* < 0.001). 18 out of 22 participants using doxycycline for PEP, and 5 out of 6 PrEP users previously had received doxycycline for targeted therapy. Therefore, most participants used pills left over from previous doxycycline treatment of STIs for PEP or PrEP (Fig. 1). Eight participants received prescriptions for doxycycline-PEP/PrEP from their general practitioner or from an STI specialist. Notably, the second most frequent mode of access to doxycycline was without a prescription by a non-official dispense by acquaintances with a healthcare profession (e.g., physicians or pharmacists). Combined with delivery by acquaintances without healthcare profession and ordering doxycycline online, use of doxycycline without a regular prescription accounted for 40% (14 of 35) of all cases.

**Table 1:** Use of doxycycline of survey participants in indicated age ranges

Age range	*n*	Never took doxycycline, *n* (%)	Doxycycline therapy, *n* (%)	Doxycycline-PEP, *n* (%)	Number of all doxycycline-PEP courses*, *n* (%)			Doxycycline-PrEP, *n* (%)	Number of all doxycycline PrEP courses*, *n* (%)		
					1 − 3*x*	4 − 10*x*	> 10*x*		1 − 3*x*	4 − 10*x*	> 10*x*
< 18	2	1 (50)	1 (50)	–	–	–	–	–	–	–	–
18–25	10	7 (70)	3 (30)	1 (10)	1 (100)	0	0	-	-	-	-
26–30	19	13 (68)	5 (26)	4 (21)	2 (50)	1 (25)	1 (25)	2 (11)	0	1 (50)	1 (50)
31–35	27	8 (30)	19 (70)	7 (26)	4 (57)	2 (29)	0	1 (4)	1 (100)	0	0
36–40	16	5 (31)	9 (56)	6 (22)	2 (33)	3 (50)	1 (17)	2 (33)	1 (50)	1 (50)	0
41–45	9	2 (22)	6 (67)	2 (22)	2 (100)	0	0	–	–	–	–
46–50	5	–	5 100)	–	–	–	–	–	–	–	–
> 50	7	2 (29)	5 (71)	2 (29)	1 (50)	1 (50)	0	–	–	–	–
n.a	1	–	–	–	–	–	–	1	1 (100)	0	0
Total	96	38 (40)	53 (55)	22 (23)	12 (55)	7 (32)	2 (9)	6 (6)	3 (50)	2 (33)	1 (17)

*Percentage rates refer to all participants who indicated to use doxycycline-PEP or PrEP within the age range

## Discussion

Our survey indicates that doxycycline-PEP/PrEP is already well known within the MSM community and that a relevant proportion of community members already uses doxycycline for STI prophylaxis. However, important limitations such as responder bias have to be taken into account. Our study included predominantly community members of a regional area. Therefore, the prophylactic use of doxycycline might be even higher in the catchment areas of large cities (e.g., Berlin, Cologne). And finally, the small number of participants might have influenced our results. Nevertheless, even with a margin of error of ± 10% the results of this small pilot study highlight a relevant use of doxycycline for STI prophylaxis and frequent modes of access to doxycycline without regular prescriptions.

The concept of prophylactic use of doxycycline needs to be critically evaluated, considering the potential development of resistance among the target pathogens (*Chlamydia* spp. and *Treponema pallidum*) as well as other STI agents like *Mycoplasma genitalium*, which has already become an emerging antibiotic-resistant pathogen [10, 11]. In addition, since resistance rates of *Neisseria gonorrhoeae* (NG) to doxycycline are already at a high level, the benefit of doxycycline for prophylaxis of NG in the German community is limited and further impact of doxycycline use on the development of increasing resistance rates of NG has to be evaluated [2, 3]. Moreover, the possible effect on development of resistance in non-target pathogens like *Staphylococcus aureus* or the gastrointestinal microbiota in general has to be considered in the context of an increased use of doxycycline. In the study of Luetkemeyer et al., no significant effects concerning resistance development were detected for *N. gonorrhea* and *S. aureus* [4]. However, the number of bacterial isolates (and genera/species) examined was relatively small. Larger studies with a more intense or longer use of doxycycline-PEP (or PrEP) may be needed to secure sufficient statistical power with regard to resistance development. Of note, previous studies on the use of doxycycline for malaria prophylaxis have shown that doxycycline use is associated with the acquisition of doxycycline/tetracycline co-resistance in intestinal bacteria [12]. Beyond that, recent studies discussed additional effects of doxycycline on resistance development of *N. gonorrhoeae* against ceftriaxone, the current first-line treatment in gonorrhea [13].

Further research is needed to evaluate the efficacy, safety, and long-term implications concerning development of resistance in a holistic approach, i.e., in the genital and extra-genital microbiota. With adequate modeling studies, we may be able to define a risk threshold (number of STIs in a given population), above which doxycycline-PEP/PrEP strategies yield to a lower doxycycline intake than repeated therapeutic cycles. By expanding our knowledge in this area, we can develop adapted strategies to address the individual sexual health needs of gay, bisexual, transgender, and queer people, ultimately contributing to the overall improvement of sexual health outcomes in this community.

## Data Availability

Access of the survey dataset can be provided on individual request.
